# On the incorrect use of *Spinella* for species of *Orostachys* subsect. *Appendiculatae* (Crassulaceae)

**DOI:** 10.3897/phytokeys.276.195585

**Published:** 2026-06-05

**Authors:** Neng Wei, Xiang-Dong Qiu, Qing-Feng Wang

**Affiliations:** 1 State Key Laboratory of Plant Diversity and Speciality Crops, Wuhan Botanical Garden, Chinese Academy of Sciences, Wuhan 430074, Hubei, China Wuhan Botanical Garden, Chinese Academy of Sciences Wuhan China; 2 Sino-Africa Joint Research Center, Chinese Academy of Sciences, Wuhan 430074, Hubei, China School of Ecology and Environment, Tibet University Lhasa China; 3 School of Ecology and Environment, Tibet University, Lhasa 850032, Xizang, China Sino-Africa Joint Research Center, Chinese Academy of Sciences Wuhan China

**Keywords:** *
Amblystachys
*, conserved type, Crassulaceae, nomenclature, *
Orostachys
*

## Abstract

Recent phylogenomic studies have consistently shown that *Orostachys* s.l. is polyphyletic and that the traditional subsections *Appendiculatae* and *Orostachys* represent distinct lineages. New generic name, *Spinella* H.-R. Lee & K.-S. Cheon was proposed to refer to species previously placed in *O.* subsect. *Appendiculatae*. We argue that this generic treatment is nomenclaturally incorrect. *Orostachys* is a conserved name with a conserved type corresponding to *O. chlorantha* (= *O. spinosa*), which is placed in subsect. *Appendiculatae*. Therefore, the name *Orostachys* must apply to the lineage traditionally treated as subsect. *Appendiculatae*, not to subsect. *Orostachys*. Consequently, *Spinella* is not the correct generic name for species of *O.* subsect. *Appendiculatae*, and should be treated as the heterotypic synonym of *Orostachys*.

## Introduction

The generic delimitation of *Orostachys* Fisch. (1809: 270) has long been controversial. Traditional classifications recognized two subsections within *O.* sect. *Orostachys*: subsect. *Appendiculatae* Ohba (1978: 160), characterized by spinose appendages at the leaf apex, and subsect. *Orostachys*, lacking such appendages (Ohba 1978). However, the two subsections have repeatedly failed to form a monophyletic group in morphological and molecular studies (Kim and Lee 1996; Mayuzumi and Ohba 2004; Gontcharova et al. 2006; Gontcharova and Gontcharov 2009; Messerschmid et al. 2020). In particular, the subsect. *Orostachys* was once proposed to merge with *Hylotelephium* H. Ohba (1977: 46) by Shaw (2017a), according to phylogenetic findings over the past two decades (Mayuzumi and Ohba 2004; Gontcharova et al. 2006; Kozyrenko et al. 2013; Nikulin et al. 2015). However, this subsection contains the original type species of the genus, *O. malacophylla* (Pall.) Fischer (1809: 274). With the purpose of stabilizing nomenclature, as well as to minimize the disruption to taxonomy of *Orostachys* and *Hylotelephium*, Shaw (2017b) proposed to conserve *Orostachys* with the new type species, *O. chlorantha* Fisch. (1809: 274) (= *O. spinosa* (L.) Sweet (1830: 225)). This proposal was later accepted by the General Committee (Wilson 2022). Therefore, the generic name *Orostachys* must follow the conserved type, i.e. the *O. chlorantha*/*O. spinosa* lineage, according to the International Code of Nomenclature for algae, fungi, and plants (Turland et al. 2025). That lineage corresponds to the traditional subsect. *Appendiculatae*. Therefore, *Orostachys* should be retained for subsect. *Appendiculatae*, not for subsect. *Orostachys*.

Recent phylogenomic studies have strengthened the case for splitting *Orostachys* s.l. Using Angiosperms353 data, Lee et al. (2025) recovered subsect. *Orostachys* as a distinct monophyletic lineage separated from subsect. *Appendiculatae* and related genera. Using Hyb-seq and plastome data, Cao et al. (2026), similarly recovered the *O. spinosa* clade (= subsect. *Appendiculatae*) and the *O. malacophylla* clade (= subsect. *Orostachys*) as separate lineages within *Orostachys* s.l. Conserquently, Cao et al. (2026) treated the subsect. *Appendiculatae* as *Orostachys* s.s., and established new genus *Amblystachys* for the subsect. *Orostachys*. However, Lee et al. (2026) described *Spinella* as a new genus segregated from *Orostachys*, and stated that it was established to accommodate taxa previously placed in *Orostachys* subsect. *Appendiculatae*, without realizing the current nomenclatural consequences of the conserved type. Instead, they designated *Spinella japonica* as the type species of the new genus and transferred nine taxa to *Spinella*. Accordingly, the treatment of Lee et al. (2026) did not follow the nomenclatural rule. Herein, we treat *Spinella* as the heterotypic synonym of *Orostachys*. Therefore, names under *Spinella* should be treated as homotypic synonyms of the corresponding names in *Orostachys*.

## Nomenclature

***Orostachys* Fisch., [Mém. Soc. Imp. Naturalistes Moscou 2: 270–274. 1809], nom. cons**.

≡ *Orostachys* sect. *Orostachys* subsect. *Appendiculatae* (Boriss.) H. Ohba, J. Fac. Sci. Univ. Tokyo, Sect. 3, Bot. 12(4): 160. 1978. Type (designated by Shaw (2017b)): *O. chlorantha* Fisch., nom. superfl. ≡ *Cotyledon spinosa* L. ≡ *Orostachys spinosa* (L.) Sweet.

= *Spinella* H.-R. Lee & K.-S. Cheon, [Korean J. Pl. Taxon., 56(1): 35–41. 2026] syn. nov. Type: *Spinella japonica* (Maxim.) H.-R. Lee and K.-S. Cheon.

## Additional information

### Artificial Intelligence (AI) use

The authors accept full responsibility for the content of the manuscript, including the disclosure of any use of AI.

No AI tools were used in the preparation of this manuscript.

### Author ORCIDs

N. Wei https://orcid.org/0000-0002-0313-3912

X.-D. Qiu https://orcid.org/0009-0007-2464-6921

Q.-F. Wang https://orcid.org/0000-0001-9143-8849

### Data availability

All of the data that support the findings of this study are available in the main text.
